# Cholinergic neural activity directs retinal layer-specific angiogenesis and blood retinal barrier formation

**DOI:** 10.1038/s41467-019-10219-8

**Published:** 2019-06-06

**Authors:** G. A. Weiner, S. H. Shah, C. M. Angelopoulos, A. B. Bartakova, R. S. Pulido, A. Murphy, E. Nudleman, R. Daneman, J. L. Goldberg

**Affiliations:** 1 Neurosciences Graduate Program, University of California, San Diego, La Jolla, CA 92037 USA; 2 Medical Scientist Training Program, University of California, San Diego, La Jolla, CA 92037 USA; 3 Spencer Center for Vision Research, Byers Eye Institute, Stanford University, Palo Alto, CA 94303 USA; 4 Department of Pharmacology, University of California, San Diego, La Jolla, CA 92037 USA; 5 Shiley Eye Institute, Department of Ophthalmology, University of California, San Diego, La Jolla, CA 92037 USA

**Keywords:** Cell biology, Developmental biology, Neuroscience, Blood-brain barrier

## Abstract

Blood vessels in the central nervous system (CNS) develop unique features, but the contribution of CNS neurons to regulating those features is not fully understood. We report that inhibiting spontaneous cholinergic activity or reducing starburst amacrine cell numbers prevents invasion of endothelial cells into the deep layers of the retina and causes blood-retinal-barrier (BRB) dysfunction in mice. Vascular endothelial growth factor (VEGF), which drives angiogenesis, and Norrin, a Wnt ligand that induces BRB properties, are decreased after activity blockade. Exogenous VEGF restores vessel growth but not BRB function, whereas stabilizing beta-catenin in endothelial cells rescues BRB dysfunction but not vessel formation. We further identify that inhibiting cholinergic activity reduces angiogenesis during oxygen-induced retinopathy. Our findings demonstrate that neural activity lies upstream of VEGF and Norrin, coordinating angiogenesis and BRB formation. Neural activity originating from specific neural circuits may be a general mechanism for driving regional angiogenesis and barrier formation across CNS development.

## Abstract (web summary)

During retinal development, waves of cholinergic neural activity play a role in retinal circuit development. Here, the authors show that this activity also contributes to layer-specific angiogenesis and formation of the blood-retinal barrier.

## Introduction

Blood vessels in the CNS have unique properties not found in peripheral vasculature. Many of these properties, such as the set of functions known as the blood–brain barrier (BBB) or blood–retinal barrier (BRB), localize to central nervous system (CNS) endothelial cells (ECs)^1,2^. CNS ECs lack fenestrations, have tight junctions, and form close associations with processes from brain parenchymal cells like microglia, pericytes, astrocytes, and neurons^3,4^. Microglia^5,6^, pericytes^7^, and astrocytes^8,9^ contribute to the function of the BBB and the patterning of blood vessels during development. Some studies have suggested that altering neural activity can have small effects on vascular patterning in the cortex, but the role of neurons, and neural activity, in the development and maintenance of CNS vascular properties has not been decisively established^10–13^.

**Fig. 1:**
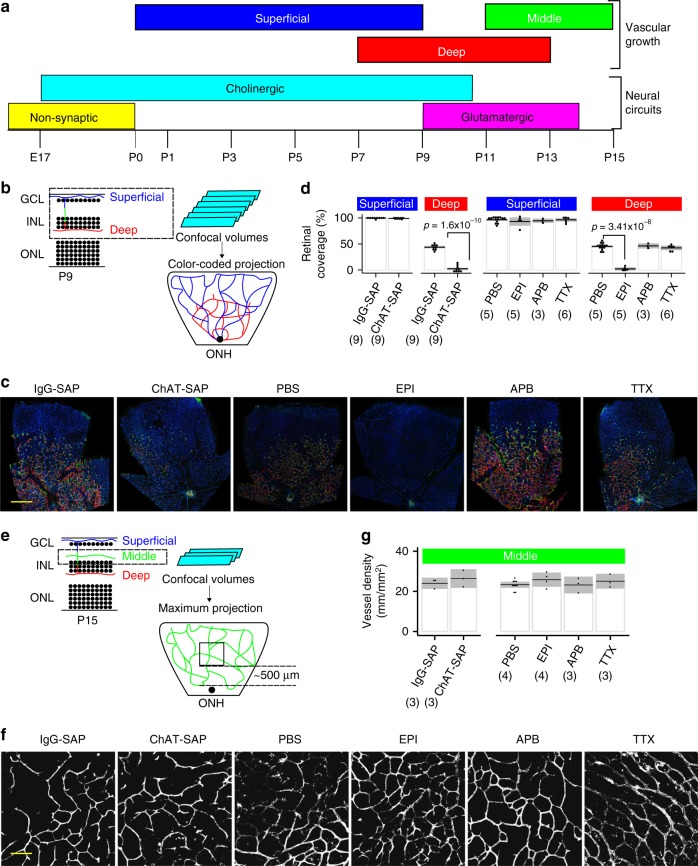
Inhibition of SAC activity selectively inhibits deep vascular plexus formation. **a** Neural circuit-specific activity overlaps with layer-specific angiogenesis in the developing mouse retina. **b** Schematic of the confocal imaging strategy for BSL-stained retinas. Confocal stacks taken at P9 were color-coded for depth so that the superficial layer is blue, penetrating vessels are green, and deep-layer vessels are red. **c** Anti-ChAT-SAP was injected intravitreally at P3 and pharmacologic inhibitors (EPI, APB, and TTX) were injected intravitreally at P3, P5, and P7 and the retinas were analyzed at P9. Scale bar is 200 µm. **d** The deep and superficial layers develop radially from the optic nerve head (ONH) and thus were quantified as the percentage of retinal surface area covered by each vascular layer. EPI and ChAT-SAP injected retinas both demonstrate a lack of deep-layer vasculature not observed in APB- or TTX-injected eyes or in control eyes (IgG-SAP and PBS). **e** Schematic of the strategy for isolating the middle layer by confocal imaging of BSL-stained retinas at P15. **f** Anti-ChAT-SAP was injected intravitreally at P11 and pharmacologic inhibitors (EPI, APB, and TTX) were injected intravitreally at P11 and P13, and the retinas were analyzed at P15. **g** The middle vascular layer develops contemporaneously at all retinal eccentricites, and thus was quantified by sampling an area near the ONH and measuring the linear amount of vessels in the middle layer per unit area of retina. None of the injected compounds had any effect on middle layer development. Scale bar is 50 µm. Statistical tests are Student’s *t* tests, two-tailed, paired when comparing control and treated eyes from the same animal, otherwise assuming unequal variances. In **d**, **g**, individual data points are plotted, dashed line is the mean, gray box is the 95% confidence interval, number of retinas analyzed is in parentheses, and all other comparisons were not significant. Number of independent samples in each group is in parentheses under the *X*-axis data label. Bandeiraea simplicifolia lectin I (BSL) staining was used to visualize vessels

In the postnatal mouse retina, waves of spontaneous neural activity mediated by a succession of neural circuits occur contemporaneously^14^ with layer-specific angiogenesis (Fig. 1a)^15^. A period of cholinergic neural activity (P0–P10) driven by starburst amacrine cell (SAC) homotypic connections and heterotypic connections to retinal ganglion cells (RGCs) overlaps with the development of the superficial and deep retinal vascular plexuses. SACs are the only cholinergic neuron in the retina^16^. Spontaneous correlated SAC activity is known to play a role in the refinement of intraretinal synaptic connections^14,17^ and eye-specific segregation in the lateral geniculate nucleus^18,19^, but no role in vascular development has been described. However, angiogenesis is thought to be driven by the relative hypoxic demand induced by tissue metabolism^20^. Since neural tissue accounts for a disproportionate amount of energy utilization in the body^21,22^, we asked whether the activity of specific neurons or circuits contribute to compartment-specific angiogenesis and BRB formation.

## Results

### A targeted toxin reduces SAC number and inhibits deep-layer angiogenesis

Anti-ChAT-SAP is a targeted toxin that selectively kills cholinergic neurons in multiple CNS regions^23–26^. In the retina, ChAT-conjugated toxins reduce SACs and block cholinergic retinal waves^27,28^, but do not affect the architecture of bipolar cell projections^29^. We reduced SACs by about 40% without affecting RGC or bipolar cell numbers (Supplementary Fig. S1A–D) by intravitreal injection of anti-ChAT-SAP at P3 and measured the extent of the superficial and deep vascular plexuses at P9 by confocal imaging (Fig. 1b, c). After SAC reduction there was a substantial reduction in deep-layer vasculature at P9 (Fig. 1d), which normally covers nearly 50% of the retinal area at this developmental age, although single vessels were occasionally present. SAC reduction did not inhibit growth of the superficial layer (Fig. 1d), which is also developing from P0 to P9, nor did SAC reduction at P11 inhibit the growth of the middle layer (Fig. 1e–g), which develops from P11 to P15 during a phase of glutamatergic retinal waves. Thus SAC reduction specifically inhibits angiogenesis of deep-layer vessels during the period of cholinergic waves.

### Cholinergic blockade specifically prevents deep-layer angiogenesis

SACs generate spontaneous activity by homotypic connections to other SACs and heterotypic connections onto a subset of RGCs^30^. Reducing SACs ablates retinal waves but does not affect the overall electrical activity of RGCs^28^. We sought to clarify the role of RGC activity versus SAC activity using pharmacological inhibitors. Tetrodotoxin (TTX) inhibits RGC action potentials but does not affect propagating cholinergic retinal waves^31^. Epibatidine (EPI) desensitizes nicotinic cholinergic receptors (nAChRs) on both SACs and RGCs and is well-known to block cholinergic waves^32–39^, allowing the independent effect of RGC action potentials and SAC activity to be established. The glutamate analog 2-amino-4-phosphonobutyrate (APB) blocks the later-appearing glutamatergic waves, and does not have any effect on cholinergic waves^40–42^. EPI had the same effect on vascular development as SAC reduction, substantially reducing deep-layer formation when injected from P3 to P9 (Fig. 1b–d), but had no effect on superficial layer formation, and no effect on middle layer formation from P11 to P15 (Fig. 1e–g). TTX had no effect on vascular development (Fig. 1b–g) suggesting the specific activity of SACs conferred this effect. APB also had no effect (Fig. 1b–g) from P3 to P9, further supporting the specific role of cholinergic activity in driving angiogenesis from P3 to P9 and consistent with the prior data on SAC reduction and consistent with the switch from cholinergic to glutamatergic waves during this later period of development.

To further address the role of SACs neural activity, we expressed the inhibitory designer receptor exclusively activated by designer drugs (Gi-DREADD) in SACs using the ChAT promoter (Supplementary Fig. S3A). Systemic administration of CNO from P3 to P9 decreased deep-layer angiogenesis, but had no effect on middle layer angiogenesis from P11 to P15 (Supplementary Fig. S3B–D). Thus SAC activity is required for normal vascularization of the deep retinal vascular layer, but not the superficial layer.

### BRB function is compromised after cholinergic disruption

**Fig. 2:**
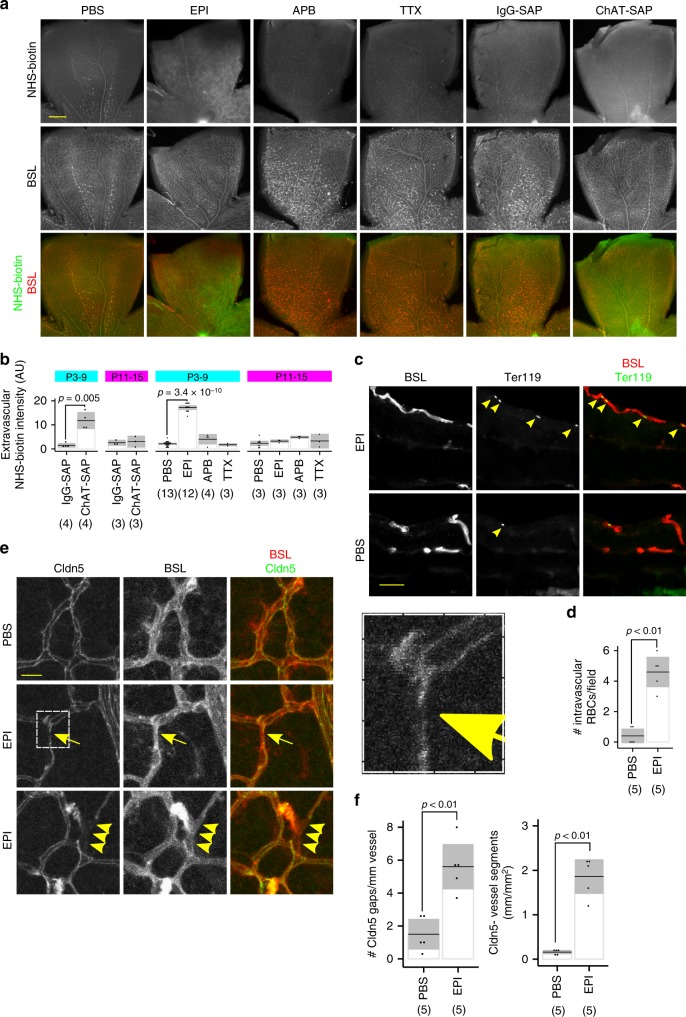
Inhibition of SAC activity from P3 to P9 leads to breakdown of the blood–retinal–barrier (BRB). **a** BRB integrity was assessed by perfusing 0.25 mg/mL NHS–biotin after SAC reduction and pharmacological inhibition of SAC activity from P3 to P9. Extravascular NHS–biotin was visualized by streptavidin-AlexaFluor 594 staining, and quantified as the amount of signal outside of the BSL-defined vasculature. Scale bar is 200 µm. **b** Quantification of extravascular NHS–biotin accumulation by fluorescence microscopy. Inhibition of SAC activity from P3 to P9, but not P11 to P15 led to increased extravascular accumulation of NHS–biotin. TTX and APB had no effect on NHS–biotin leakage. **c** Intraretinal hemorrhage was examined by anti-Ter119 immunostaining, which identifies red blood cells (RBCs). No intraretinal extravascular RBC was observed in any EPI or PBS injected eye. Scale bar is 50 µm. **d** Quantification of intravascular RBCs. There was an increase in intravascular RBCs after transcardial perfusion in EPI eyes. A similar effect of anti-ChAT-SAP was observed (Supplementary Fig. S5). **e** Cldn5 expression was examined by immunofluorescent staining. Altered Cldn5 expression correlated with BRB dysfunction after EPI injection. Breaks in continuous Cldn5 junctional strands were observed (middle row, arrow). Hatched box is magnified at right showing an example of a junctional gap, which was rarely observed in control retinas. Some short vessel segments in EPI eyes had lost Cldn5 expression completely (bottom row, arrowheads). Scale bar is 15 µm. **f** EPI treatment increased the number of Cldn5 gaps and the amount of Cldn5-negative vasculature. In **b**, statistical tests are Student’s two-tailed *t* tests, paired for comparing control and treated eyes from the same animal; in **d**, **f** statistical tests are the Mann–Whitney *U* test. Number of independent samples in each group is in parentheses under the *X*-axis data label

We investigated the function of the BRB as measured by NHS–biotin permeability under the same sets of conditions: SAC reduction, pharmacological blockade of cholinergic activity, and chemogenetic inhibition of SACs from P3 to P9 and P11 to P15. SAC reduction, EPI injection and Gi-DREADD activation in SACs led to increased barrier permeability at P9, but not at P15 or with TTX (Fig. 2a, b, Supplementary Fig. S3E), indicating that SAC activity is required for regulating BRB properties only during the period of cholinergic waves. Increased barrier permeability can be secondary to retinal hemorrhage, but no extravascular red blood cells (RBCs) were observed after EPI injection, although there was an increase in the number of intravascular RBCs remaining after transcardial perfusion (Fig. 2c). Along with the observed central distribution of NHS–biotin leakage, this suggests that even the superficial layer vessels in EPI-injected retinas are not well-perfused by the circulatory system. To determine why there was increased BRB permeability we examined the expression of claudin-5 (Cldn5), which is critical to form the paracellular barrier between endothelial cells^43^. Expression of Cldn5 was disrupted after EPI injection as evidenced by gaps in the normally smooth junctional strands, and by areas of Cldn5-negative vessels (Fig. 2e, f). EPI injection did not alter Cav1 or Plvap expression (Supplementary Fig. S6).

### Neural activity regulates angiogenic and barriergenic signaling

**Fig. 3:**
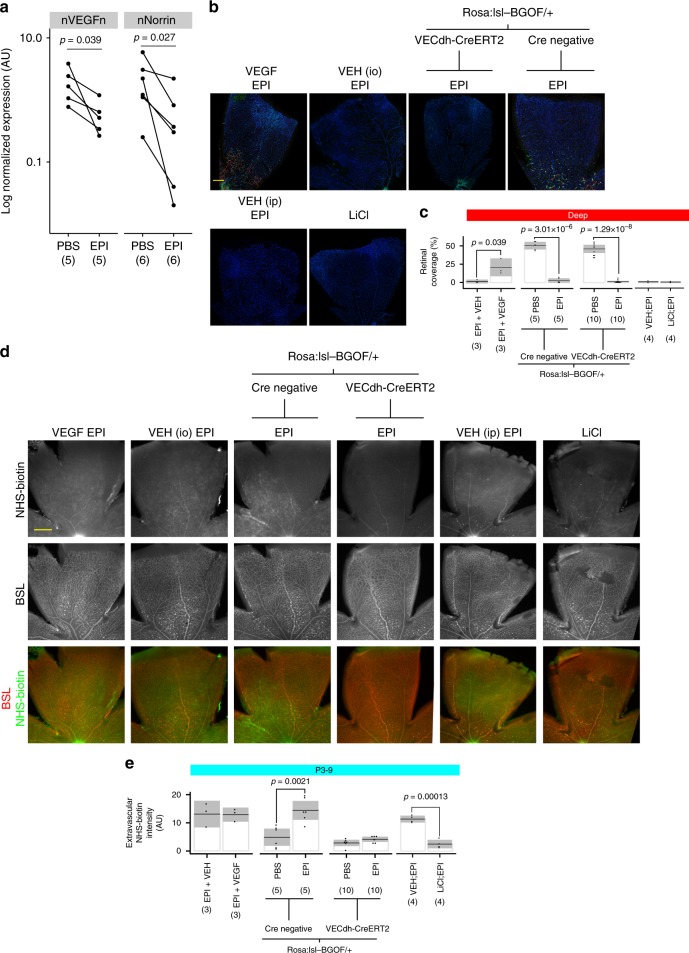
VEGF limits angiogenesis and Norrin limits barrier function independently in the context of SAC activity blockade. **a** Expression of VEGF and Norrin was measured by Western blot after inhibition of SAC activity with EPI from P3 to P9. VEGF and Norrin expression are both decreased after activity blockade. **b**–**e** Recombinant VEGF was injected or beta-catenin activity was increased by expression of a stabilized form of beta-catenin (BGOF) in endothelial cells by removal of a *lox-stop-lox* sequence by Cre under the VE-Cadherin promoter (CreERT2) or systemic lithium chloride (LiCl) administration. The effects on deep vascular plexus growth (**b**, **c**) and NHS–biotin permeability (**d**, **e**) were examined. Scale bar is 200 µm in each. **d** VEGF increased deep-layer angiogenesis, but enhancing beta-catenin signaling had no effect. **f** VEGF did not further increase vascular permeability but enhancing beta-catenin signaling prevented the increase in permeability induced by SAC activity blockade. In **a**, **c**, **e**, statistical tests are Student’s two-tailed *t* tests, paired for comparing control and treated eyes from the same animal. Number of independent samples in each group is in parentheses under the *X*-axis data label

Together, defective angiogenesis and barriergenesis after inhibition of SAC activity led us to hypothesize that pro-angiogenic and pro-barriergenic signaling may be decreased in the retina after SAC blockade. Vascular endothelial growth factor (VEGF) is known to be important in developmental and pathological angiogenesis^44^. Wnt signaling, mediated by Muller glia-derived Norrin, has been shown to be important for both retinal angiogenesis and BRB barriergenesis^45,46^. The BRB is comprised of multiple functions, many of which have been shown to be Wnt-dependent^45–52^.Vasculature restricted to the superficial vascular plexus and Cldn5 dysregulation are features observed in Norrin knockout mice^53^. We tested whether inhibition of SAC activity affected the expression of VEGF and Norrin, and found both were decreased (Fig. 3a).

VEGF can increase vascular permeability and drive angiogenesis and Norrin can decrease vascular permeability and is also required for retinal-specific angiogenesis^53,54^. We next sought to clarify the contribution of VEGF and Norrin to the vascular abnormalities observed after inhibition of cholinergic activity. VEGF was co-injected with EPI and found to restore some deep-layer angiogenesis (Fig. 3b, c), but did not have any additional effect on increasing the permeability of the BRB (Fig. 3d, e), suggesting that EPI alone fully permeabilizes the BRB. To determine the role of Norrin/beta-catenin signaling, we expressed a stabilized form of beta-catenin (BGOF) under the control of the vascular endothelial cadherin promoter (VECdh-CreERT2). We also injected lithium chloride (LiCl) systemically, which has been shown to stabilize beta-catenin via inhibition of glycogen synthase kinase 3 beta (GSK3beta)^55^. Neither BGOF nor LiCl increased deep vascular plexus growth (Fig. 3b, c), but both prevented the increase in BRB permeability after inhibition of SAC activity (Fig. 3d, e). Together, these data show that VEGF and Norrin are the critical limiting factors for angiogenesis and barriergenesis respectively, in the context of SAC activity blockade.

### Inhibition of cholinergic activity reduces pathological neovascularization

**Fig. 4:**
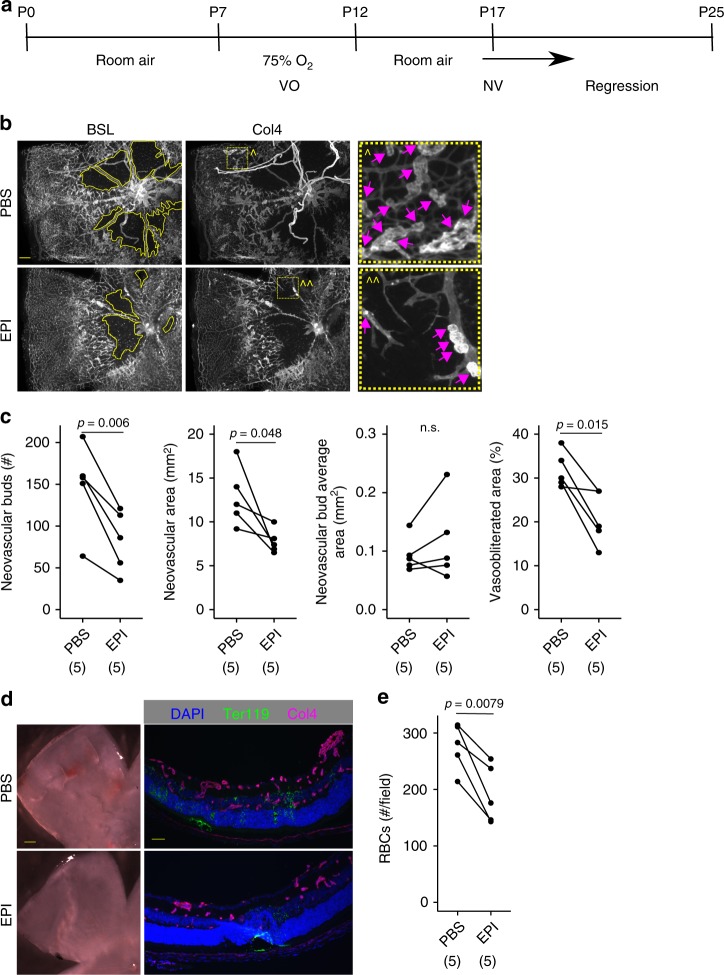
Inhibition of SAC activity decreases neovascularization and hemorrhage in OIR. **a** Mouse pups were raised on room air until P7, when they were transferred to a 75% O_2_ chamber. At P12 the pups were removed from the hyperoxic chamber and placed back into room air. P7–P12 is the vaso-obliterative (VO) phase when previously established vessels regress. From P12 to P17 the retina undergoes profound pathological neovascularization (NV), and then from P17 to P25 the neovessels gradually regress. We inhibited cholinergic activity from P12 to P17 with every other day EPI injections and examined the retinas at the peak of neovascularization. **b** BSL was used to visualize the amount of vaso-obliteration and anti-Collagen IV (Col4) to visualize the bright basement membrane of preretinal neovascular buds. Zoomed in box shows examples of neovascular buds from the areas indicated by the hatched boxes. Scale bar is 200 µm. **c** The number of neovascular buds, total area of neovascularization, and area of vaso-obliteration all decreased after SAC inhibition, but there was no change in the average size of each neovascular bud (n.s. is not significant). **d** Intraretinal hemorrhage was visible by brightfield imaging of freshly dissected retinas, and by anti-Ter119 staining of fixed retinal sections. Scale bar is 200 µm on the left, 50 µm on the right. **e** SAC inhibition decreased the number of RBCs in the retina. In **c**, **e**, statistical tests are Student’s two-tailed *t* tests, paired for comparing control and treated eyes from the same animal. Number of independent samples in each group is in parentheses under the *X*-axis data label

We next asked whether similar activity-dependent angiogenesis and barriergenesis is disrupted in a disease model of the developing retinal vasculature. Retinopathy of prematurity (ROP) is a disease that afflicts premature infants subject to the high oxygen concentrations needed to sustain blood O_2_ saturation. A common feature of ROP as well as other retinopathies of adulthood diseases is the presence of abnormal neoangiogenic vessels that leak, hemorrhage, and physically distort the retina, leading to loss of vision. We asked whether cholinergic signaling plays a role in pathological neovascularization observed during ROP and tested this hypothesis using the oxygen-induced retinopathy (OIR) mouse model of ROP^56^. Inhibiting SACs during the neovascular phase from P12 to P17 (Fig. 4a) with intravitreal EPI injections decreased the area of vaso-obliteration (VO, Fig. 4b), the amount of neovascularization (Fig. 4b), and the amount of hemorrhage in the retina (Fig. 4d, e). This demonstrates that cholinergic signaling is a critical component driving pathogenic retinal neoangiogenesis.

## Discussion

In summary, these data point to three important conclusions. First, neural activity derived from a specific circuit underlies both regional angiogenic drive and barrier formation in this CNS vasculature. Although the metabolism of neural tissue may account for a disproportionate amount of total body energy utilization, several aspects of this work shed further light on the relationship between specific neural activity and angiogenesis. In particular, it is not generic basal metabolism of neurons but rather the synaptic and downstream activity originating from a specific neural subtype during a select period of retinal development that drives oxygen consumption, angiogenesis, and barriergenesis. SACs may be well positioned to account for significant energy utilization in the retina as their dendritic fields overlap more extensively than those of other retinal neurons^57^, and dendrites contribute more than other neural compartments to metabolic demand^58^. In fact, we observed that SAC activity and retinal activity driven by SACs accounts for nearly 50% of retinal oxygen consumption in early postnatal isolated retinas (Fig. S3F, G). Because the effect of EPI on cholinergic activity is transient, the inhibition of angiogenesis was transient; eyes injected with EPI from P3 to P9 subsequently recovered the normal trilaminar vascular network by P21 (Fig. S2D). This is consistent with prior observations that EPI-induced inhibition of cholinergic waves is transient, with resumption of cholinergic waves after blockade is lifted, and subsequent delayed initiation of the late phase glutamatergic waves^59^. Thus neural activity may be a generic stimulus for triggering angiogenesis in the CNS, but this dependence may be restricted to specific circuits and CNS regions at different periods in development.

Second, these data show that neural activity lies upstream of pro-angiogenic and probarriergenic signaling, and temporally coordinates the action of these pathways. VEGF and Wnt are both required for CNS angiogenesis^54^, and Wnt specifically for BRB function^60^ but the independent contributions of each to vessel formation and barrier induction are not understood. Analysis of knockout animals is complicated by the fact that VEGF is increased after Norrin knockout in mice^61^. These animals have a disrupted BRB, but is it because EC’s lack Wnt signaling or because VEGF is increased? Here we show the functions of VEGF and Norrin are uncoupled in the context of diminished developmental neural activity such that VEGF controls vascular outgrowth and Norrin turns on BRB properties. How might neural activity coordinate such a signal? Prior work has shown that layer-specific angiogenesis is due to VEGF derived from specific cell types^62^. In that case, amacrine and horizontal cell-derived VEGF drives intermediate plexus formation. However, it is also known that Muller glia sense neurotransmitter spillover from amacrine cell synapses^63^, thus neural activity may be driving VEGF production in nonneuronal cell types as occurs, for example, with astrocytes producing the VEGF that drives superficial layer angiogenesis^20^. These data further suggest the intriguing possibility that the microenvironmental cues needed to guide vessel development are present even without the stimulus of neural activity necessary to produce the pro-angiogenic factors.

Third, we have implicated a role for cholinergic signaling in pathological neoangiogenesis that occurs in vascular retinopathies. We studied this question in OIR and found that cholinergic blockade reduced neovascularization. This implicates cholinergic signaling as a central regulator of angiogenesis in development and disease, though the source of acetylcholine in OIR is not clearly identified. We showed that cholinergic blockade inhibits deep-layer angiogenesis, yet in OIR, neovascularization occurs in the superficial layer. The mechanism by which normal developmental vascularization and pathologic neovascularization are related is unclear, but these data suggest that further study of cholinergic activity may help uncouple these mechanisms. Further work should define the role of cholinergic signaling and the activity of other neurotransmitter-specific circuits in neovascular diseases of adulthood, such as wet age-related macular degeneration and proliferative diabetic retinopathy, as well as investigate the specific circuits responsible for angiogenesis in different CNS compartments. Given the range of pharmacological modulators known to act on receptors and enzymes of the cholinergic pathway, these results extend this broad category of therapeutics to potential use in neovascular disease.

## Methods

### Animals

Wild-type animals were C57/BL6 mice purchased from Envigo. Homozygous ChAT-IRES-Cre knock-in mice (ChatCre) animals were purchased from The Jackson Laboratory (strain 006410). Heterozygous R26-LSL-Gi-DREADD (RGi/+) animals were purchased from The Jackson Laboratory (strain 026219). ChatCre homozygotes were crossed to RGi/+ animals, and littermate R+/+ animals were used as controls. All animal experiments were performed in accordance with national guidelines and UCSD IACUC guidelines. Experimental/surgical procedures were performed to minimize animal stress and the number of animals used.

### Statistical methods

Statistical methods are detailed in each figure. In general, for comparing control and treated retinas from the same animal, paired, two-tailed Student’s *t* tests were used to account for batch-to-batch variability between experiments (images are presented without any contrast enhancement), otherwise a two-tailed Student’s *t* test assuming unequal variances was used. For counting RBCs and quantification of Cldn5 integrity, the Mann–Whitney *U* test was used. Number of animals used in the statistical analysis, as well as exact *p* values for those tests that reached a significance level of *p* < 0.05 are reported in each figure.

### Inhibition of cholinergic activity

Intravitreal injections were done at P3, P5, and P7 for experiments during the cholinergic period, or at P11 and P13 for experiments during the glutamatergic period. For the OIR model, injections were done at P13 and P15. In each case, the injection procedure was similar. Animals were anesthetized using isoflurane flowing through a rodent facemask. Isoflurane was 1–3%, flow 100 mL/min, and the procedure lasted approximately 20 min per animal. After reflex checks, a small cut was made through the eyelid and the lid pulled back to expose the eye. After P13 the eyes were typically open and the eyelids did not need to be cut open. Approximately, 0.5–1.0 uL of solution was injected using pulled glass micropipettes attached to a picospritzer III in P3–P9 pups, 1.5–2.0 uL in P11–P15 pups. Typically, 20–40 ms duration pulses at 30 PSI were used, but this was adjusted as needed to inject the appropriate amount. The needle was left in the eye for 30 s after injection and withdrawn slowly to minimize leakage. The eyelid was sutured for P3–P9 animals and covered with antibiotic ointment for animals at every age. One eye was injected with the experimental compound and the other eye was injected with a vehicle control. Drug concentrations and vehicles were epibatidine (Sigma E1145) 1 mM in phosphate-buffered saline (PBS), APB (Sigma A1910) 1 mM in PBS, TTX (Tocris 1069) 1 mM in citrate buffer, Rabbit anti-ChAT-SAP (Advanced Targeting Systems IT-42) 0.12 mg/mL in PBS. The control for ChAT-SAP was an untargeted Rabbit anti-IgG-SAP (Advanced Targeting Systems IT-35) 0.12 mg/mL in PBS.

### CNO injections

Clozapine-n-oxide was purchased from Tocris (4936) dissolved at 20 mM in sterile water, and stored in the dark at −20 degrees. Prior to injection, CNO was diluted in sterile saline and injected to a final concentration of 0.5 mg/kg. Two daily intraperitoneal injections were performed, spaced 12 h apart.

### Retina collection for vascular analysis

Pups were killed and the whole eyes were fixed in 4% PFA in 1× PBS at room temperature for 10 min, then transferred to 1× PBS on ice. The retinas were dissected out and fixed in methanol at −20° overnight.

### Retina collection for barrier analysis

Pups were deeply anesthetized using a ketamine/xylazine mixture and then sequentially transcardially perfused with ~10–15 mL of 0.25 mg/mL EZ-Link Sulfo-NHS–Biotin in PBS and ~10–15 mL of 4% PFA in PBS. The whole eyes were fixed in 4% PFA in 1× PBS at room temperature for 10 min, then transferred to 1× PBS on ice. The retinas were dissected out and postfixed in 4% PFA for 45 min to 1 h then washed in PBS and stored in PBS plus azide at 4° in the dark. Results of the NHS–biotin uptake assay were validated against a sodium fluorescein uptake assay (Supplementary Fig. S4).

### Immunofluorescence

Whole-mount fixed retinas were washed in PBS then blocked for 30 min in 0.2% bovine serum albumin, 5% serum, and 0.3% Triton X-100, then incubated in primary antibody in blocking buffer overnight at 4° with gentle rocking. Retinas were then washed in 0.3% Triton X-100 three times, and incubated with secondary antibodies in 0.3% Triton X-100 for 2–4 h at room temperature, protected from light with gentle rocking. They were then washed in 0.3% Triton X-100 twice, 1x PBS twice, and then mounted on slides with Prolong Gold Antifade Reagent. Primary antibodies used were rabbit anti-Cldn5 (Thermo Fisher Scientific 34-1600, 1:500), goat anti-collagen 4 (SouthernBiotech 1340-08, 1:500), and rat anti-Ter119 (abcam ab91113,1:250). Secondary antibodies used were goat anti-rabbit Alexa Fluor 488 (Thermo Fisher Scientific A-11034, 1:1000), goat anti-rat donkey anti-goat Alexa Fluor 488 (Thermo Fisher Scientific A27012, 1:1000), donkey anti-rabbit Alexa Fluor 488 (Thermo Fisher Scientific A-21206, 1:1000), goat anti-rabbit Alexa Fluor 594 (Thermo Fisher Scientific A-11037, 1:1000), and donkey anti-goat Alexa Fluor 594 (Thermo Fisher Scientific A-11058, 1:1000). Bandeiraea simplicifolia lectin (BSL) was biotinylated griffonia simplicifolia lectin I (Vector Laboratories B-1205, 1:250) or fluorescein conjugated (FL-1201). Streptavidin was streptavidin conjugated to Alexa Fluor 594 (Thermo Fisher Scientific S32357, 1:1000).

### Confocal and epifluorescence imaging

Confocal imaging was done on a Zeiss LSM 710. A 20 × /0.8 NA air objective was used. Images were acquired at 1 airy unit resolution with 4× undersampling of pixels in X–Y and 2× undersampling in Z. Excitation lasers were 488 nm (for Alexa Fluor 488 and fluorescein isothiocyanate (FITC)), 561 nm (for Alexa Fluor 594), and 633 nm (for Alexa Fluor 647). Emissions were collected as separate tracks over wavelength bands exclusive with other mutually excited fluorophores. Epifluorescence images were collected on an Axio Imager D2 (Carl Zeiss) with a 5× Fluor, 0.25 NA or 20× Plan-Apochromat, 0.8 NA objective, using a digital camera (Axiocam HRm, Carl Zeiss). AxioVision software was used to acquire images; Fiji (ImageJ) and Inkscape were used for image processing and analysis.

### Vascular image analysis

Confocal volumes were stitched using Zeiss’ Zen software using a strictness of 0.9. Because the stacks were often not completely flat, they were then rotated in ImageJ using the TransformJ plugin so the superficial layer vasculature was in the same plane across every stack in the image. The image was then depth-coded in ImageJ by coloring the stacks according to Z-position along a blue–green–red axis. Retinal area was quantified in ImageJ by manually selecting the extent of the flat mount. Coverage of a vascular layer was quantified by manually selecting the area of the retina covered by that layer and then dividing by the whole retinal area. For analyzing the middle layer, a ~425 µm × 425 µm z-stack was taken ~500 µm away from the center of the retina. Vessels were traced by hand, skeletonized in ImageJ using the Skeletonize plugin, and analyzed for length and branch points using the same plugin.

### BRB image analysis

Epifluorescence images were acquired by focusing on the superficial layer vasculature. The whole retina was imaged then stitched together using the MosaicJ plugin in ImageJ or the Zen Software package. Intravascular regions were defined by BSL-FITC staining, and used to create a mask of the NHS–biotin image. The masked NHS–biotin image represents extravascular NHS–biotin accumulation. To account for differences in absolute brightness of NHS–biotin brightness from experiment to experiment, the extent of the retina was defined and the average extravascular NHS–biotin intensity per pixel was divided by the background (nonretinal) intensity per pixel to yield the extravascular NHS–biotin in arbitrary units.

### Angiogenesis and barrier rescue experiments

Epibatidine was injected to inhibit cholinergic activity from P3 to P9 as previously described. In wild-type animals EPI was injected into both eyes. For VEGF rescue, recombinant human VEGF-165 (R&D Systems, 293-VE-010) dissolved in PBS was co-injected with EPI in one eye at 38 µg/mL. Total injection volume was the same for both eyes. In transgenic BGOF animals, EPI was injected as previously described with PBS as control. At P7 20 µL of 2 mg/mL tamoxifen (Sigma T5648) dissolved in corn oil was injected intraperitoneally. For LiCl rescue, LiCl (Sigma L7026, 30 mg/kg) or saline was injected intraperitoneally once per day at P7, P8, and P9.

### Measurement of oxygen consumption rate

OCR was measured on a Seahorse XFe96 Flux Analyzer. One millimetre punches from isolated retinas were placed in the bottom of Seahorse XFe96 spheroid plates. Dissections and incubations were done in DMEM 5030 supplemented to 20 mM glucose, 10 mM HEPES, 2 mM glutamine, and 5 mM pyruvate.

### Oxygen-induced retinopathy

At P7 a litter of mice (pups and mom) were introduced in a sealed Biospherix chamber linked to an oxygen line. The O_2_ is brought up to 75%. Mice were observed daily without opening the chamber. At P12, 5 days later, mice were removed from the chamber and placed back in a normoxic environment.

### Western blots

Eyes from mice were removed after CO_2_ euthanasia and flash frozen with liquid nitrogen and stored in −80 °C. After defrosting, the retinas were dissected and homogenized by sonication in 90 µL of cold ddH_2_0 with cOmplete Protease Inhibitor cocktail (CO-RO ROCHE) and 10 µL 10× RIPA Lysis Buffer (20–188 EMD MILLIPORE). Samples were centrifuged at 10,000×*g* for 10 min at 4 °C, and the supernatants collected. Total protein concentration was determined using a BCA assay (Pierce BCA Protein Assay Kit 23225). Totally, 30–40 g of protein was loaded on a 4–20% tris-glycine gel (Thermo Fisher Scientific XP04202) and transferred to an activated 0.2 µm PVDF membrane for immunoblotting. The membranes were saturated with TBS 1×, 0.05% Tween-20, and 5% nonfat dry milk for 1 h at room temperature, then incubated overnight at 4 °C with mouse anti-Norrin (1:300, R&D Systems MAB3014), mouse anti-VEGF C-1(1:300, Santa Cruz Biotechnology sc-7269), or rabbit anti-GAPDH (1:2000, Cell Signaling Technology #2118). All membranes were washed with TBS-Tween between incubations. The membranes were then saturated with peroxidase-conjugated goat anti-mouse or anti-rabbit (1:3000, Abcam, ab6789 and ab6721) secondary antibodies for 3 h at room temperature, and revealed with SuperSignal West Pico Chemiluminescent Substrate (Thermo Fisher Scientific 34080) and imaged with myECL Imager (Thermo Fisher Scientific). Secondary only controls were done to ensure that probing mouse tissue homogenates with an anti-mouse primary did not detect endogenous IgG. Stripping to ensure equal loading was done with ReBlot Plus Strong Antibody Stripping Solution (EMD Millipore 2504). All quantifications were done with ImageJ densitometry analysis.

### BREC proliferation assay

Bovine retinal microvascular endothelial cells (BRECs) were maintained in fibronectin-coated plates (1 μg/cm^2^). The growth medium was low glucose DMEM supplemented with 10% bovine calf serum, 5 ng/ml bFGF and 10 ng/ml hVEGF_165_. BRECs were seeded in 96-well plates (no coating) in low glucose DMEM supplemented with 10% bovine CS, 2 mM glutamine, and antibiotics (growth medium), at a density of 1000 cells per well in 200 μl volume. VEGF165 was added at the indicated final concentrations. Cells were incubated with Alamar Blue for 4 h. Fluorescence was measured at 530 nm excitation wavelength and 590 nm emission wavelength. The experiments were repeated at least three times.

## Supplementary information

Supplementary Information

## Data Availability

The data that support the findings of this study are available from the corresponding author upon reasonable request.
