# Supplementary material for: Cholinergic neural activity directs retinal layer-specific angiogenesis and blood retinal barrier formation
Source: Nat Commun. 2019 Jun 6;10:2477. doi: 10.1038/s41467-019-10219-8 (PMC6554348; doi:10.1038/s41467-019-10219-8)
Supplement: Supplementary file 1 — Supplementary Information [file 41467_2019_10219_MOESM1_ESM.pdf]

Supplementary Information

For Weiner et al., “Cholinergic neural activity directs retinal layer-specific angiogenesis and blood-retinal barrier formation.”

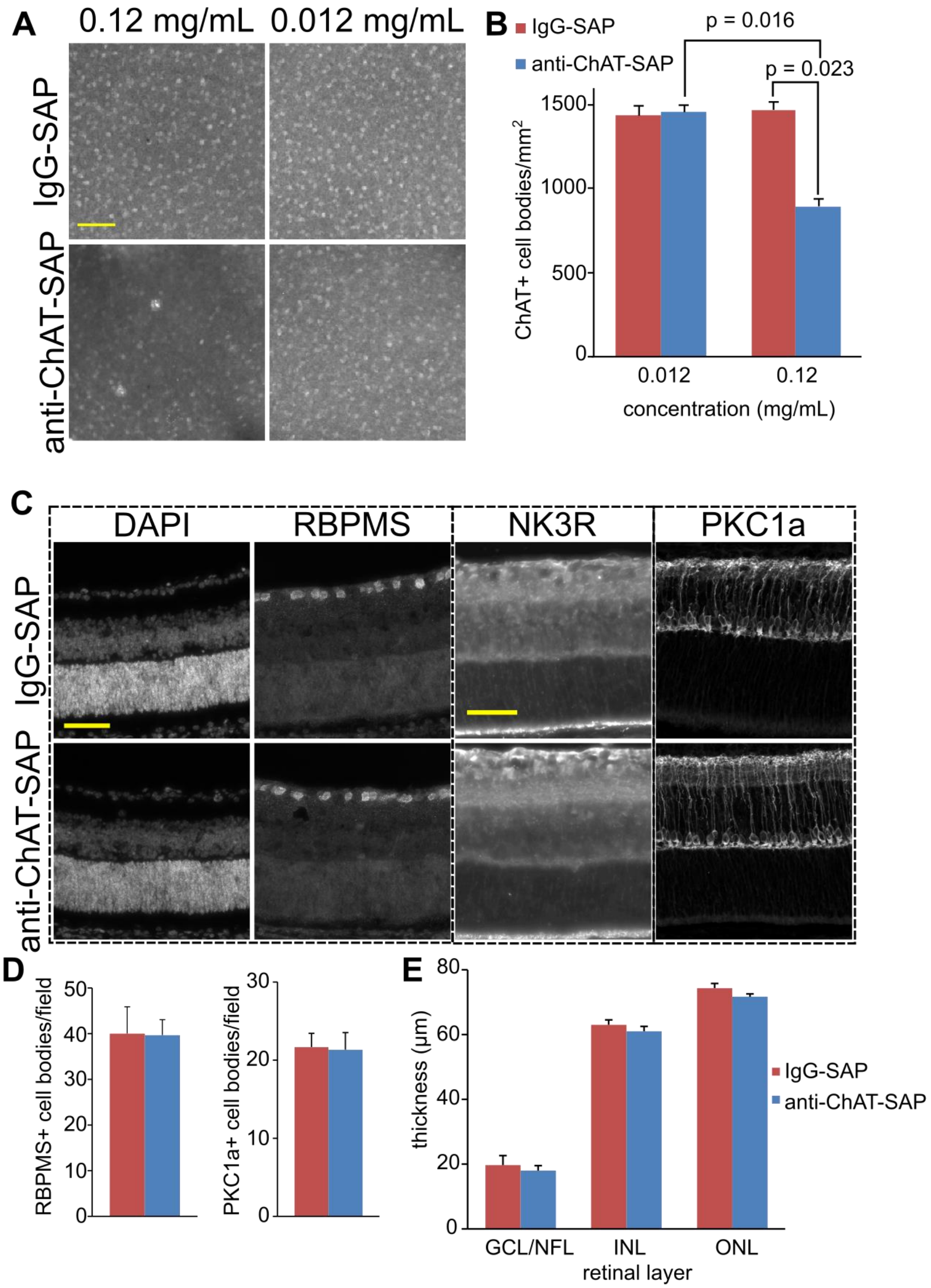

Figure S1. The targeted toxin anti-ChAT-SAP reduces SACs without disrupting normal retinal development and is not toxic to other neurons. (A) Immunofluorescence staining with an anti-ChAT antibody at P9 after injection of anti-ChAT-SAP or the control untargeted IgG-SAP at P3. Scale bar is 100 µM. (B) 0.12 mg/mL injected toxin significantly reduced the number of ChAT+ cell bodies, while 0.012 mg/mL had no effect. (C) Immunofluorescence staining with anti-RBPMS, a marker specific for retinal ganglion cells (RGCs), anti-NK3R, a marker for OFF-

cone bipolar cells, and anti-PKC1-alpha, a marker for rod bipolar cells, at P9 after injection of 0.12 mg/mL anti-ChAT-SAP at P3. DAPI staining shows the laminar structure of the nuclei. Scale bars are 50 µm. Sections taken within 200 µm of optic nerve head. Hatched line indicates eyes from different animals. (D) There was no reduction in the number of RBPMS+ cell bodies in the ganglion cell layer, no reduction in PKC1-alpha+ cell bodies in the INL, and (E) no change in the thickness of the retinal nuclear lamina. Statistical tests are Student's t-test, paired between eyes of the same animal. Error bars are SEM. All other comparisons were not significant.

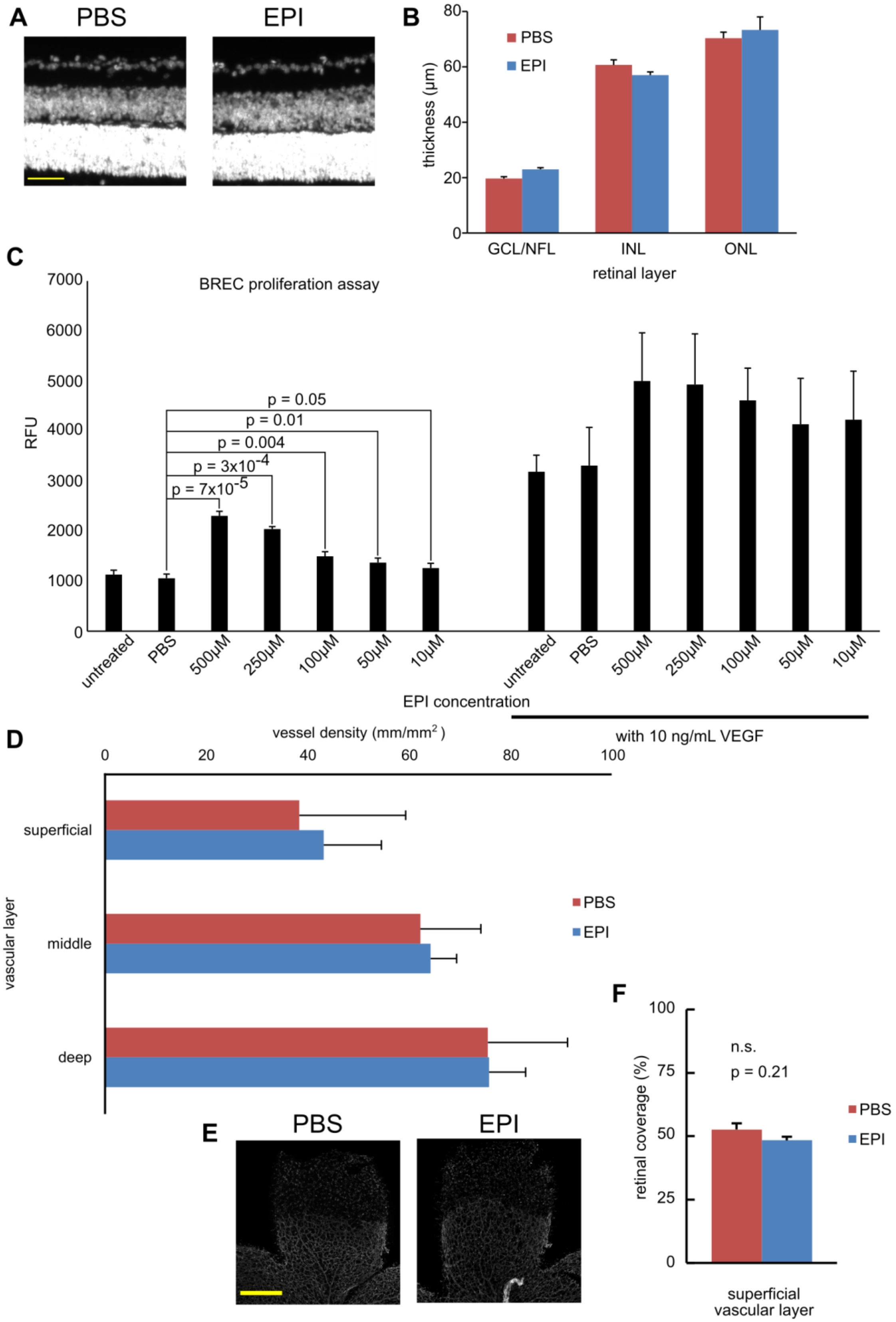
A
PBS
EPI
B
80
60
40
20
0
thickness (µm)
PBS
EPI
GCL/NFL
INL
ONL
retinal layer
C
BREC proliferation assay
7000
6000
5000
4000
3000
2000
1000
0
RFU
p = 0.05
p = 0.01
p = 0.004
p = 3x10-4
p = 7x10-5
untreated
PBS
500µM
250µM
100µM
50µM
10µM
untreated
PBS
500µM
250µM
100µM
50µM
10µM
EPI concentration
with 10 ng/mL VEGF
D
vessel density (mm/mm2)
0
20
40
60
80
100
superficial
middle
deep
vascular layer
PBS
EPI
E
PBS
EPI
F
100
75
50
25
0
retinal coverage (%)
n.s.
p = 0.21
PBS
EPI
superficial
vascular layer

Figure S2. Epibatidine (EPI) does not disrupt normal retinal development and is not toxic to endothelial cells. (A) DAPI staining shows the laminar structure of the nuclei at P9 after 1 mM EPI injections at P3, P5, and P7. Scale bar is 50 µm. Sections taken within 200 µm of optic nerve head. (B) The thicknesses of the GCL, INL, and ONL were not altered by EPI treatment. (C) Primary bovine retinal endothelial cells (BRECs) were cultured in the presence or absence of 10 ng/mL VEGF and various concentrations of EPI. EPI resulted in an increase in BREC proliferation without VEGF, demonstrating that it is not directly toxic to endothelial cells. (D) EPI was injected at P3, P5, and P7 and then the vasculature was examined at P28 by BSL staining and confocal imaging (as in Fig. 1). The vasculature had completely recovered it's normal trilaminar structure. (E) EPI was injected at P3 and the superficial layer was examined at P5 bu BSL staining and confocal imaging (as in Fig. 1). Scale bar is 500 µm. (F) The superficial layer was not transiently delayed by EPI injection. Statistical tests are unpaired Student's t-test assuming unequal variances. Error bars are SEM. All other comparisons were not significant.

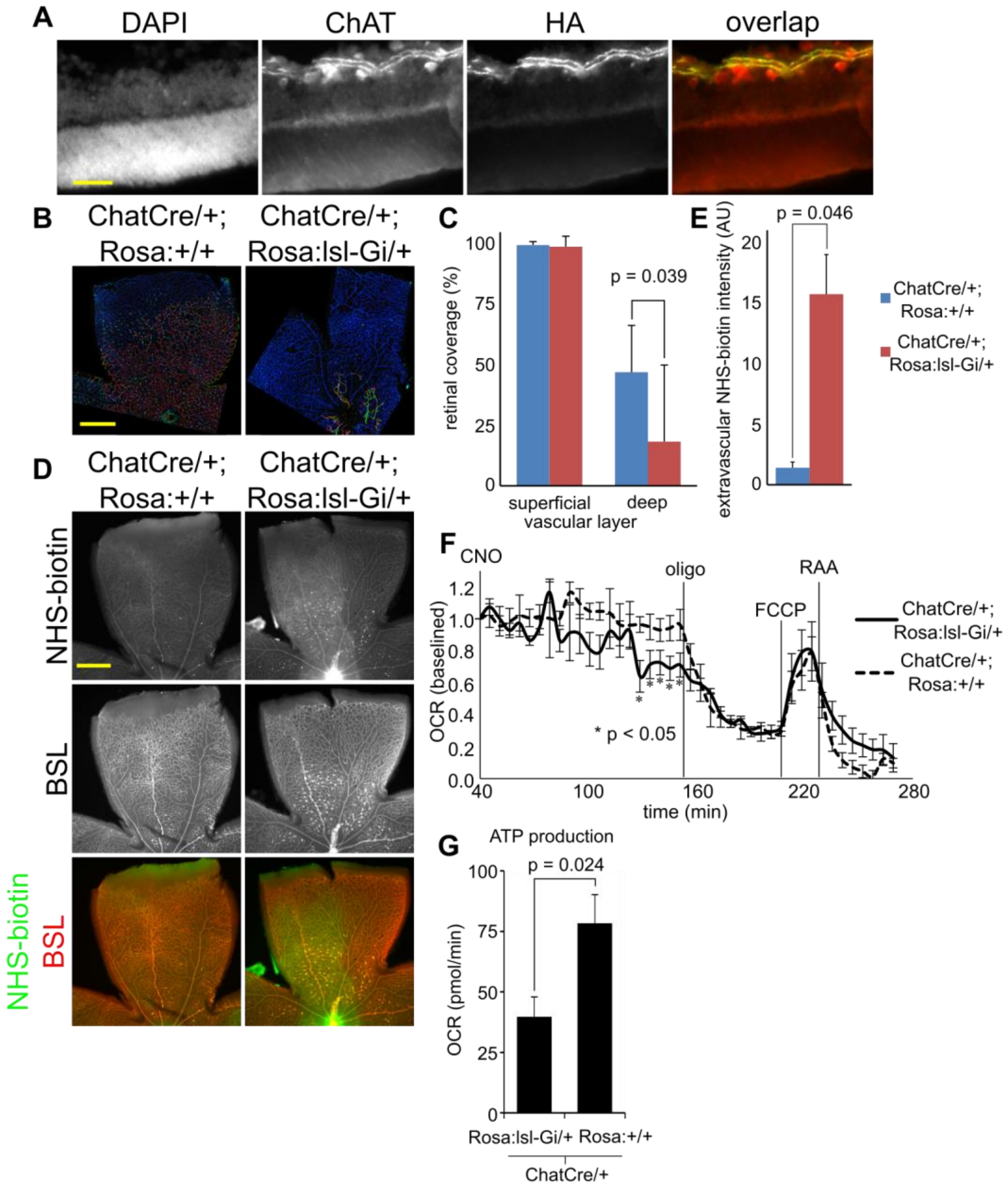

Figure S3. An inhibitory DREADD under the ChAT promoter is expressed in SACs and can inhibit whole retinal metabolism. (A) Immunofluorescence double-labeling with goat anti-ChAT (red channel in overlap) and rabbit anti-HA (green channel in overlap). HA is tagged onto the N-terminus of the DREADD Gi receptor, which is shown to be expressed in SACs, the only ChAT-expressing neuron in the retina. Scale bar is 50 µm. (B) 0.5 mg/kg CNO was injected twice daily from P5 to P9. Retinas were collected at P9. Immunofluorescence staining with BSL and depth-coded confocal projections are shown. Scale bar is 200 µM. (C) Activating the inhibitory receptor in SACs decreased deep layer angiogenesis. Scale bar is 200 µM. (D) BRB integrity was assessed by perfusing 0.25 mg/mL NHS-biotin after CNO-induced Gi inhibition of SAC activity from P3 to P9. Extravascular NHS-biotin was visualized by streptavidin-AlexaFluor 594 staining, and quantified as the amount of signal outside of the BSL-defined vasculature. (E)

CNO injection in ChatCre/+;Rosa:lsl-Gi/+ but not ChatCre/+;Rosa+/+ animals led to increased extravascular accumulation of NHS-biotin. (F) Oxygen consumption rate (OCR) was measured in isolated retinal punches using a Seahorse XFe96 analyzer before and after injections of clozapine-N-oxide into the well. Baseline period prior to CNO injection is not shown. Every measurement is expressed relative the baseline (last measurement before CNO injection into the port). ~2 hours after CNO injection there was a significant decline in total retinal OCR in Gi-DREADD expressing retinas compared to genetic controls. (G) ATP production, which is OCR just prior to oligomycin injection minus the lowest OCR recorded during the oligomycin period. ATP production declined almost 50% in the Gi-DREADD expressing retinas compared to genetic controls. Statistical tests are unpaired Student’s t-test assuming unequal variances. Errors bars are SEM. All other comparisons were not significant. No other differences in mitochondrial function were detected.

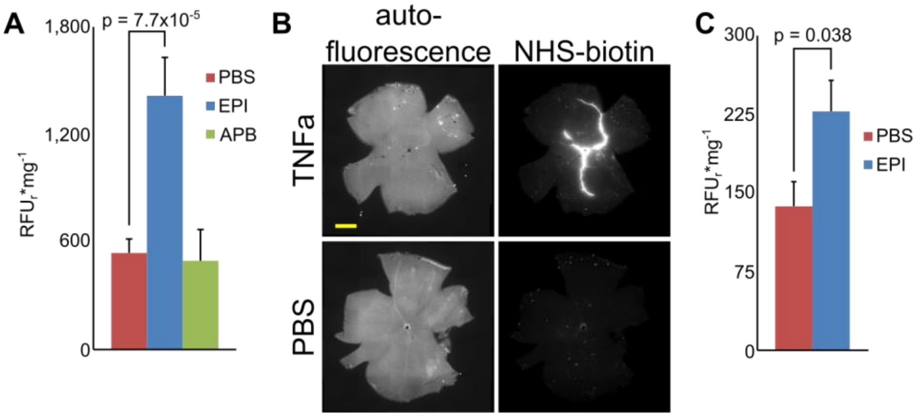

Figure S4. Blood-retinal barrier (BRB) assay. To validate the BRB integrity quantitation by NHS-biotin perfusion previously described, we also performed a sodium fluorescein uptake assay. Briefly, 10% (w/v) sodium fluorescein was injected IP, 2.5 µL per gram mouse, allowed to circulate for 10 minutes, then serum was collected and retinal tissue was harvested. The soluble fraction was extracted by trichloroacetic acid precipitation and fluorescein concentration was measured on a plate reader and compared to a standard curve. Measurements are expressed relative to wet retinal weight. (A) PBS, APB, or EPI were injected at P3, P5, and P7 and the fluorescein uptake assay was performed at P9. There was significantly more fluorescein in the retina after EPI injected compared to PBS, APB showed no difference. (B) Immunofluorescence images of streptavidin-Alexa Fluor 594 stained retinas after tumor necrosis factor alpha (TNFa) intravitreal injections and NHS-biotin perfusion. TNFa is known to disrupt the BRB, and this is shown here by leakage of perfused NHS-biotin into the extravascular space. (C) Fluorescein uptake was also increased after TNFa injection. Pharmacologic inhibitors were injected at P11 and P13 and the animals were perfused with NHS-biotin at P15. Statistical tests are paired Student’s t-test. Error bars are SEM. All other comparisons were not significant.

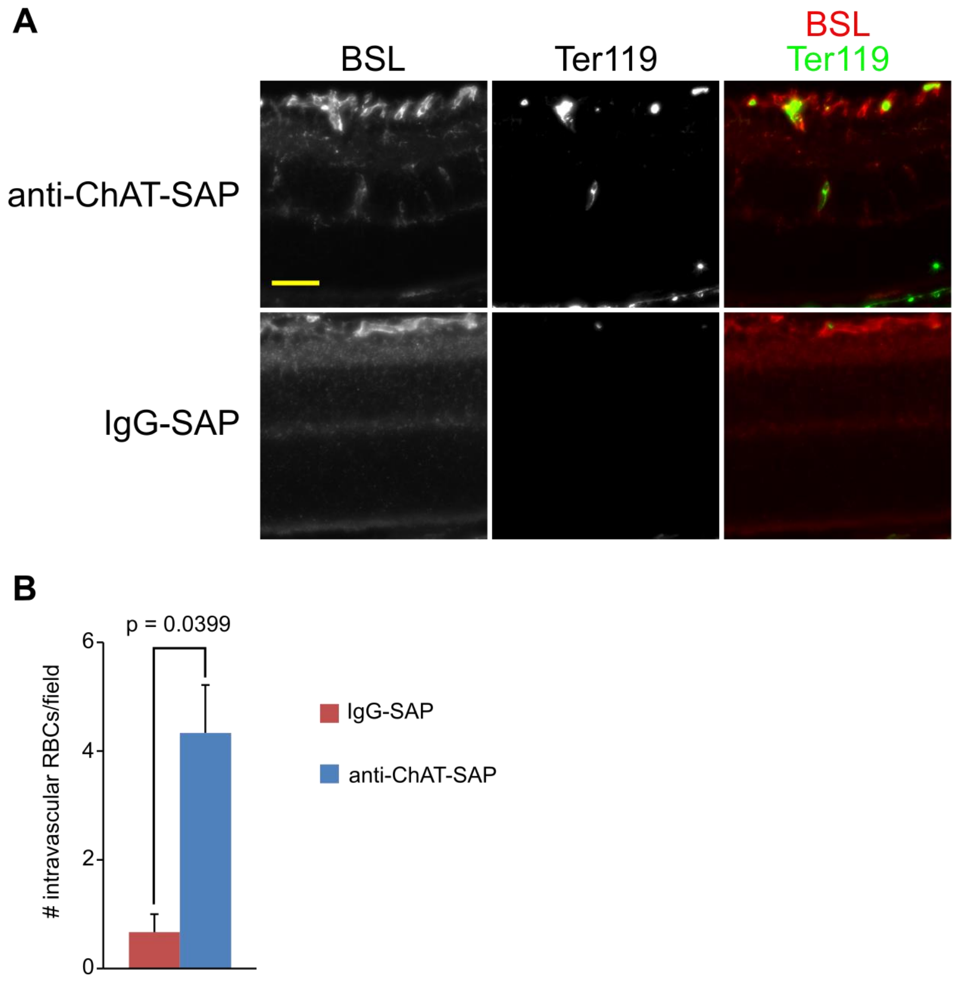

Figure S5. Anti-ChAT-SAP increases vascular retention of RBCs. Anti-ChAT-SAP was injected to diminish SACs at P3 and retinas were collected and analyzed at P9. (A) Intraretinal hemorrhage was examined by anti-Ter119 immunostaining, which identifies red blood cells (RBCs). No intraretinal extravascular RBC was observed in any injected eye. Scale bar is 50 µm. (D) There was an increase in intravascular RBCs after transcardial perfusion in anti-ChAT-SAP treated eyes. Statistical test is the Mann-Whitney U test.

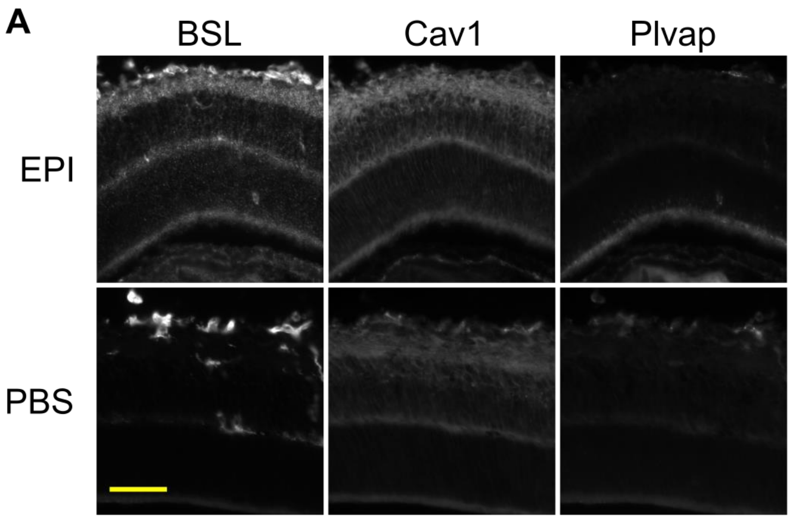

Figure S6. EPI does not alter the expression of the BRB-related genes Cav1 and Plvap. EPI was injected to silence cholinergic waves at P3, P5, and P7 and retinas were collected and analyzed at P9. (A) Immunofluorescence labeling with BSL to highlight blood vessels, anti-Cav1, and anti-Plvap antibodies. Although Cav1 appeared diffusely elevated in the retina, expression in the vasculature was not markedly different. There was no change in Plvap expression. Scale bar is 50 µm.
